# Dihydrotanshinone I Inhibits the Proliferation and Growth of Oxaliplatin-Resistant Human HCT116 Colorectal Cancer Cells

**DOI:** 10.3390/molecules27227774

**Published:** 2022-11-11

**Authors:** Mengge Wang, Yusen Xiang, Ruyu Wang, Lijun Zhang, Hong Zhang, Hongzhuan Chen, Xin Luan, Lili Chen

**Affiliations:** Shanghai Frontiers Science Center of TCM Chemical Biology, Institute of Interdisciplinary Integrative Medicine Research, Shanghai University of Traditional Chinese Medicine, Shanghai 201203, China

**Keywords:** Dihydrotanshinone I, CRC, oxaliplatin-resistant, virtual screening, SHP2

## Abstract

Oxaliplatin (OXA) is a first-line chemotherapeutic drug for the treatment of colorectal cancer (CRC), but acquired drug resistance becomes the main cause of treatment failure. Increasing evidence has shown that some natural components may serve as chemoresistant sensitizers. In this study, we discovered Dihydrotanshinone I (DHTS) through virtual screening using a ligand-based method, and explored its inhibitory effects and the mechanism on OXA-resistant CRC in vitro and in vivo. The results showed that DHTS could effectively inhibit the proliferation of HCT116 and HCT116/OXA resistant cells. DHTS-induced cell apoptosis blocked cell cycle in S and G_2_/M phases, and enhanced DNA damage of HCT116/OXA cells in a concentration-dependent manner. DHTS also exhibited the obvious inhibition of tumor growth in the HCT116/OXA xenograft model. Mechanistically, DHTS could downregulate the expression of Src homology 2 structural domain protein tyrosine phosphatase (SHP2) and Wnt/β-catenin, as well as conventional drug resistance and apoptosis-related proteins such as multidrug resistance associated proteins (MRP1), P-glycoprotein (P-gp), Bcl-2, and Bcl-xL. Thus, DHTS markedly induces cell apoptosis and inhibits tumor growth in OXA-resistant HCT116 CRC mice models, which can be used as a novel lead compound against OXA-resistant CRC.

## 1. Introduction

Chemotherapy is the most important treatment for patients with advanced colorectal cancer (CRC) [1]. The FOLFOX regimen is a classic first-line treatment regimen for CRC, mainly composed of oxaliplatin (OXA), calcium folate, and fluorouracil [2,3]. OXA-based chemotherapy is one of the most commonly used therapeutic strategies after surgery [4]. OXA-resistance is a key contributor to treatment failure and tumor progression [5]. Tumor-related molecular mechanisms of OXA-resistance reported mainly include [6,7]: (1) overexpression of ATP binding box (ABC) transporter; (2) DNA damage repair; (3) block cells G_2_ phase arrest; (4) enhance the anti-apoptosis ability of cells. Therefore, finding the underlying mechanism and the means to reverse/sensitize OXA resistance are urgently needed for the treatment of CRC patients.

Various active ingredients from Chinese medicine can inhibit chemotherapy-induced drug resistance in CRC [8]. Studies have shown that curcumin can reverse OXA-resistance in OXA-resistant cell line HCT116/OXA [9]. Spica Prunellae extract enhanced fluorouracil (FU) sensitivity in 5-FU-resistant human CRC HCT-8/5-FU cells [10]. Resveratrol increased the sensitivity of HCT116 and CT26 cells to cetuximab [11]. Dihydroisotanshinone I (DT) was also reported to induce apoptosis in CRC cell lines HCT116 and HT-29, and significantly inhibited tumor growth in a HCT116 xenograft nude mouse model [12]. Dihydrotanshinone I (DHTS) inhibits the formation of osteosarcoma by decreasing both the transcriptional activity and the total protein expression of β-catenin [13]. However, whether DHTS can inhibit OXA-resistant CRC and the underlying molecular mechanism is unknown.

SHP2 is the first oncogenic protein identified in the protein tyrosine phosphatase family encoded by PTPN11 [14]. As a promising anticancer drug target, SHP2 plays a key role in many cancer-related signaling pathways, such as Ras-Raf-ERK, PI3K-Akt, and JAK-STAT and PD-1/PD-L1 pathways [15]. It has been shown that SHP2 is an upstream protein of β-catenin [16,17], which is required for activating the promitogenic/oncogenic Wnt signaling pathway. The prevalent feature in the progression of CRC is oncogenic mutations in the Wnt/β-catenin signaling pathway [18]. The Wnt/β-catenin signaling pathway promotes chemotherapeutic drug resistance through up-regulation of drug-resistant protein P-gp [19]. Therefore, whether inhibition of SHP2 and Wnt/β-catenin-associated signaling pathways can overcome OXA-induced CRC drug resistance is worthy of in-depth investigation.

In this study, we found DHTS, the active ingredient of *Salvia miltiorrhiza* with anti-CRC activity based on ligand-based virtual screening and cell-based assay. Interestingly, we observed its inhibitory effect on OXA-resistant HCT116/OXA cells and a HCT116/OXA nude mouse xenograft model. The results showed that DHTS significantly inhibited the proliferation of HCT116/OXA resistant strains, induced cell apoptosis, blocked cell cycle in S and G_2_/M phases, and enhanced DNA damage in a concentration-dependent manner. DHTS could also reduce the expression of MRP1 and P-gp, anti-apoptotic proteins Bcl-2 and Bcl-xL, as well as SHP2 and Wnt/β-catenin signaling related proteins. Taken together, our findings are the first to reveal that the antitumor activity of DHTS in vitro and in vivo against OXA-resistant CRC is associated with the expression of SHP2 and Wnt/β-catenin pathway protein.

## 2. Results

### 2.1. Identification of Natural Compounds with the Anti-CRC Activity Based on Virtual Screening and CCK-8 Assay

**Figure 1:**
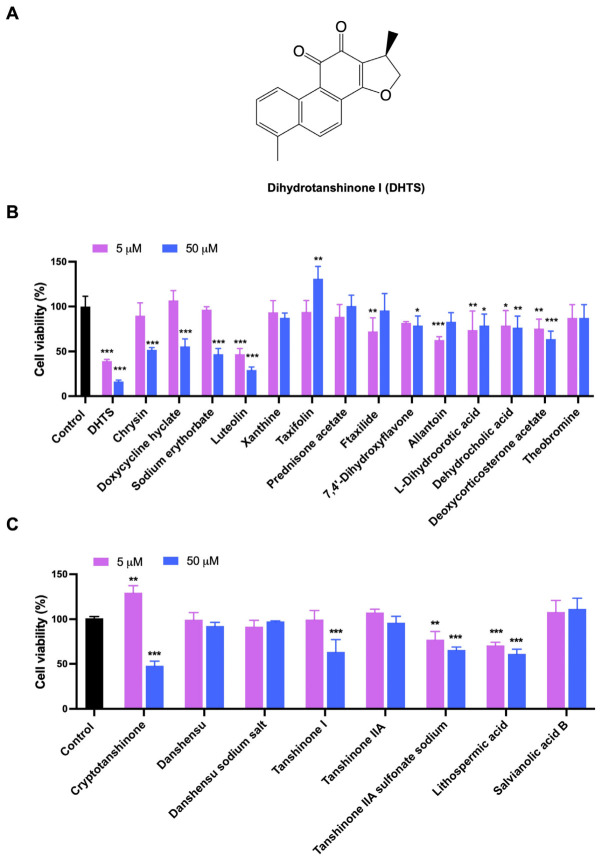
Evaluation for the growth inhibition of the test compounds in HCT116 cells. (**A**) The chemical structure of DHTS; (**B**) the inhibitory activities of 15 compounds from LBVS; (**C**) the inhibitory activities of 8 compounds from *Salvia miltiorrhiza*. HCT116 cells were treated with the test compounds at 5 and 50 μM for 24 h by CCK-8 assay (compared to the control group, * *p* < 0.05, ** *p* < 0.01, *** *p* < 0.001).

Based on LBVS, a total of 48 compounds were screened and only 15 compounds (Table S1) were used to determine their inhibitory effects on HCT116 cell proliferation. The inhibitory activities of these compounds against the proliferation of HCT116 cells were detected by CCK-8 method. The results showed that both DHTS and Luteolin greatly inhibited the cell viability of HCT116 cells, while the inhibition rate of DHTS (Figure 1A and Figure S1) exceeded 50% and 80% at 5 and 50 μM after 24 h treatment, exhibiting the strongest inhibitory effect on the proliferation of HCT116 cells (Figure 1B). Since DHTS is derived from *Salvia miltiorrhiza*, we next purchased 8 other compounds (Table S2) from *Salvia miltiorrhiza* to detect their effects on cell proliferation in HCT116 cells to find compounds with stronger inhibitory activities. As shown in Figure 1C, Cryptotanshinone resulted in more than a 50% inhibition rate in HCT116 cells at 50 μM, while the other 7 compounds showed less than 50% inhibition. Therefore, DHTS has the strongest inhibitory effect on HCT116 cell proliferation.

### 2.2. DHTS Inhibits the Growth of HCT116 Cells

**Figure 2:**
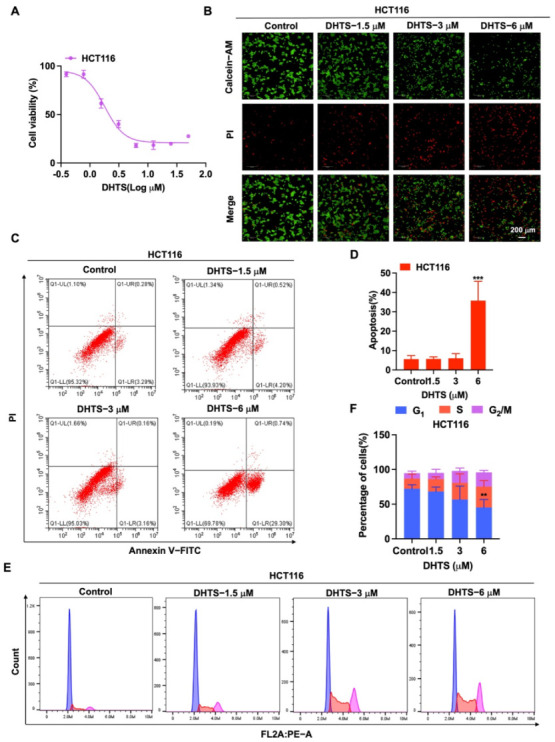
DHTS inhibited proliferation and apoptosis of HCT116 cells. (**A**) Cells proliferation assay of HCT116 cells after DHTS treatment at different concentrations for 24 h by CCK-8 assay; (**B**) cells proliferation assay of HCT116 cells after DHTS treatment at 1.5, 3, and 6 μM for 24 h by Calcein-AM/PI staining; (**C**–**F**) HCT116 cells were treated with DHTS at 1.5, 3 and 6 μM for 24 h. Apoptosis and cell cycle distribution of HCT116 cells were detected by flow cytometry (compared to the control group, ** *p* < 0.01, *** *p* < 0.001).

To determine the inhibition of DHTS against HCT116 cells, CCK-8 assays and Calcein-AM/PI staining were performed with various concentrations of DHTS. The IC_50_ value of DHTS was 2.06 ± 0.24 μM in HCT116 cells (Figure 2A). The fluorescent probes Calcein-AM and PI were used to differentiate between living and dead cells in HCT116 cells. The results showed that 6 μM DHTS resulted in a drastic reduction in living cells stained with calcein (green), and an increase in the dead cells stained with PI (red) compared with the low-concentration group (3 and 1.5 μM) and the untreated group (Figure 2B). It has been reported that apoptosis is one of the cell death patterns in multicellular organisms [20]. To estimate the effect of DHTS on apoptosis, flow cytometry analysis using double-staining with Annexin V-FITC/PI was performed in HCT116 cells. As shown in Figure 2C,D, the total apoptotic rate of HCT116 cells gradually increased with the increase of DHTS concentration. To analyze the cellular mechanism of growth inhibition of DHTS in HCT116 cells, the cell cycle distribution was evaluated by flow cytometry. The result showed the percentage distribution of the cells treated with DHTS (6 μM) was 45.27 ± 11.36%, 30.00 ± 28.81%, and 20.60 ± 2.95% at the cell cycle G_1_, S, and G_2_/M phases, respectively, compared to 72.13 ± 5.77%, 14.69 ± 6.43%, and 7.85 ± 2.92% in the untreated cells (Figure 2E,F). Therefore, these results showed that DHTS suppresses the growth of HCT116 cells.

### 2.3. Construction and Biological Characterization of OXA-Resistant Human CRC Cell Line HCT116/OXA

Based on the above results that DHTS can inhibit the proliferation and growth of HCT116 cells, we intended to determine whether this compound has the inhibitory effect on OXA-resistant CRC cells. Therefore, we constructed the OXA-resistant cell line HCT116/OXA and further investigated the effect of DHTS on the proliferation and growth of HCT116/OXA cells.

**Figure 3:**
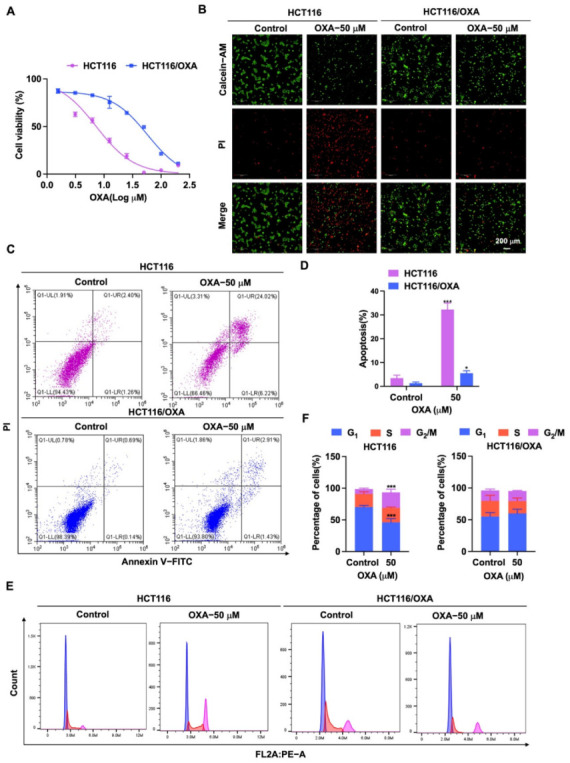
Effect of OXA on the proliferation and growth of HCT116 and HCT116/OXA cells. (**A**) In CCK-8 experiment, HCT116 and HCT116/OXA were treated with different concentrations of OXA for 48 h; (**B**) OXA (50 μM) was applied to HCT116 and HCT116/OXA cells for 48 h, and Calcein-AM/PI staining was performed for live/dead cells; (**C**–**F**) HCT116 and HCT116/OXA cells were treated with OXA (50 μM) for 48 h to detect cell apoptosis and cell cycle progression (compared to the control group, * *p* < 0.05, *** *p* <0.001).

First, HCT116/OXA cells were established by the low concentration gradient method. Then, we verified the biological characteristics of HCT116/OXA cells. HCT116 and HCT116/OXA cells were treated with different concentrations of OXA, and the IC_50_ values were 8.03 ± 1.98 and 60.88 ± 4.58 μM, respectively (Figure 3A). The resistance index was 7.58 times (IC_50_ ratio of OXA against HCT116/OXA and HCT116). Through Calcein-AM/PI staining, we found that OXA had significantly stronger inhibitory effect on HCT116 cells compared to HCT116/OXA cells (Figure 3B). Since the molecular mechanism of OXA-resistant tumors is associated with apoptosis and cycle arrest [6,7], further flow cytometry experiments were performed. The results of the apoptosis assay showed that OXA significantly enhanced apoptosis in HCT116 cells compared with HCT116/OXA cells (Figure 3C,D). The cell cycle analysis result showed the percentage distribution of HCT116 cells treated with OXA (50 μM) was 46.10 ± 5.94%, 22.77 ± 1.33%, and 24.63 ± 5.03% at the cell cycle G_1_, S, and G_2_/M phases, respectively, compared to 70.30 ± 2.46%, 20.70 ± 3.45%, and 7.64 ± 1.45% in the untreated cells (Figure 3E,F), which suggests that OXA caused apoptosis and G_2_/M cell cycle arrest in HCT116 cells. However, treatment with OXA (50 μM) in HCT116/OXA cells did not greatly change the percentage distribution of the cells in G_1_, S, and G_2_/M phases (Figure 3E,F). Therefore, these results indicated that OXA had no significant inhibitory effect on the growth of HCT116/OXA cells.

**Figure 4:**
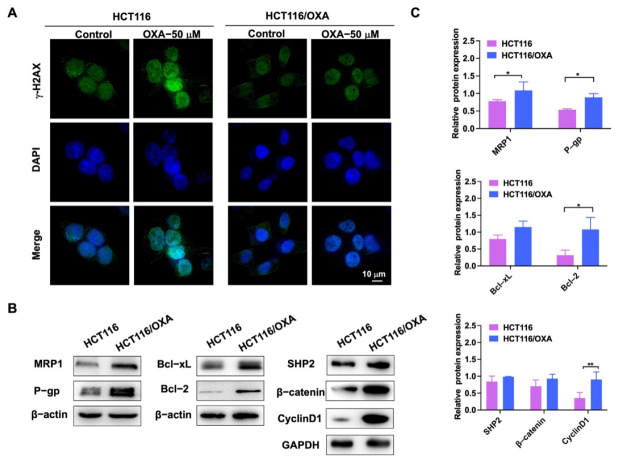
Immunofluorescence and western blot experiments verified the biological characteristics of OXA-resistant strain HCT116/OXA. (**A**) The immunofluorescence analysis of nuclear foci for γ-H2AX expression induced by OXA in parent and resistant cells after 24 h exposure to OXA; (**B**) expression of MRP1, P-gp, Bcl-2, Bcl-xL, SHP2, β-catenin, and Cyclin D1 proteins in HCT116 and drug-resistant strain HCT116/OXA; (**C**) quantitative analysis of western blot in (**B**) (compared to the control group, * *p* < 0.05, ** *p* < 0.01).

OXA binds to DNA in tumor cells to form cross-linked structures, resulting in DNA replication damage [5]. γ-H2AX was used as a marker of DNA damage in immunofluorescence experiments [7]. After OXA was applied to the parent HCT116 cells, it showed stronger DNA damage compared with the drug-resistant strain (Figure 4A). Western blot experiments were used to detect the different proteins in HCT116 and HCT116/OXA cells. The expression levels of drug-resistant proteins MRP1 and P-gp, anti-apoptotic proteins Bcl-2 and Bcl-xL, SHP2, β-catenin, and Cyclin D1 were increased in HCT116/OXA cells compared with HCT116 cells (Figure 4B,C). Therefore, these results demonstrated that we successfully constructed an OXA-resistant strain.

### 2.4. DHTS Inhibits the Proliferation of HCT116/OXA Cells

**Figure 5:**
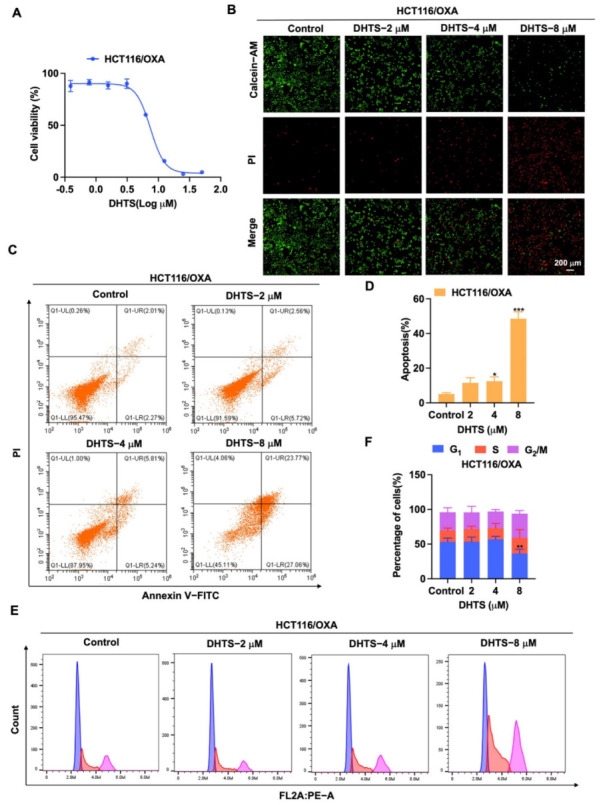
DHTS inhibits proliferation and apoptosis of HCT116/OXA cells. (**A**) Cells proliferation assay of HCT116/OXA cells after DHTS treatment at different concentrations for 48 h by CCK-8 assay; (**B**) cells proliferation assay of HCT116/OXA cells after DHTS treatment at 2, 4, and 8 μM for 48 h by Calcein-AM/PI staining; (**C**–**F**) HCT116/OXA cells were treated with DHTS at 2, 4, and 8 μM for 48 h. Apoptosis and cell cycle distribution of HCT116/OXA cells were detected by flow cytometry (compared to the control group, * *p* < 0.05, ** *p* < 0.01, *** *p* < 0.001).

For the exploration of the inhibition of DHTS on proliferation of HCT116/OXA cells, we used CCK-8 assays and Calcein-AM/PI staining to determine its inhibitory activity in HCT116/OXA cells. As shown in Figure 5A, DHTS has the obvious inhibitory effect on proliferation in HCT116/OXA cells with the IC_50_ of 6.59 ± 0.53 μM. In Calcein-AM/PI staining experiments, DHTS could significantly reduce the viability of HCT116/OXA cells (Figure 5B). To further investigate whether the inhibition of cell proliferation by DHTS was related to apoptosis, we examined the pro-apoptotic effect of DHTS in HCT116/OXA cells using flow cytometry. The results showed that DHTS could induce apoptosis of CRC-resistant cells in a dose-dependent manner (Figure 5C,D). It is well known that reduced proliferation of cancer cells is closely associated with cell cycle arrest [21]. To further investigate the growth inhibition mechanism of DHTS on HCT116/OXA cells, we examined the cell cycle distribution by flow cytometry. It was shown that the percentage distribution of HCT116/OXA cells treated with DHTS (8 μM) was 36.43 ± 6.16%, 22.67 ± 11.72%, and 34.83 ± 4.34% at the cell cycle G_1_, S, and G_2_/M phases, respectively, compared to 53.33 ± 5.25%, 16.4 ± 3.44%, and 26.2 ± 6.44% in the untreated cells (Figure 5E,F), which suggests that DHTS can significantly inhibit HCT116/OXA cells at S and G_2_/M phases. These results indicate that DHTS inhibits the growth of HCT116/OXA cells.

**Figure 6:**
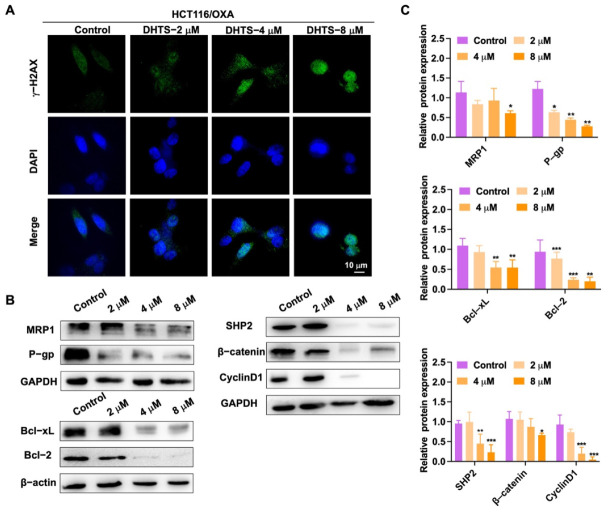
DHTS suppresses the growth of H116/OXA cells in vitro. (**A**) HCT116/OXA cells were treated with DHTS at 2, 4, and 8 μM for 48 h by immunofluorescence; (**B**) effects of DHTS on different proteins in drug-resistant strain HCT116/OXA; (**C**) quantitative analysis of western blot in (**B**) (compared to the control group, * *p* < 0.05, ** *p* < 0.01, *** *p* <0.001).

To detect DNA damage in HCT116/OXA cells by DHTS, immunofluorescence experiments were performed. In Figure 6A, the experimental results showed that DNA damage in HCT116/OXA cells could be enhanced with the increase of DHTS concentration. We further detected a change in drug-resistant-related proteins. As demonstrated in Figure 6B, C, DHTS significantly inhibited drug-resistant proteins MRP1 and P-gp, as well as anti-apoptotic Bcl-2 and Bcl-xL, and reduced the expression of β-catenin upstream proteins SHP2 and downstream proteins Cyclin D1. Therefore, DHTS can inhibit proliferation and apoptosis in HCT116/OXA cells.

### 2.5. DHTS Inhibits HCT116/OXA Tumor Growth In Vivo

**Figure 7:**
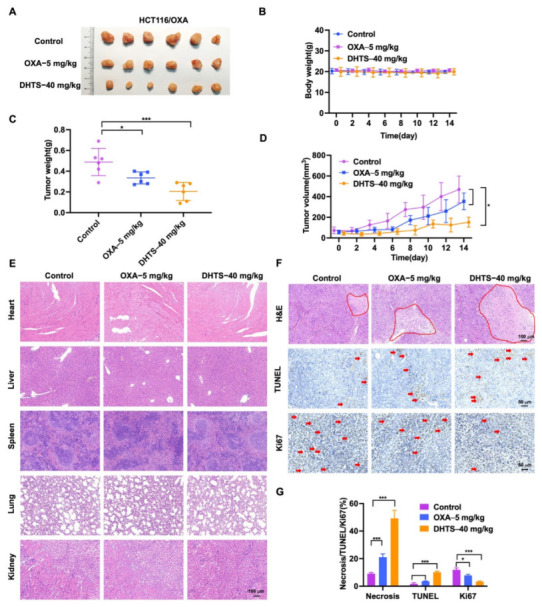
DHTS inhibits tumor growth in OXA-resistant HCT116 CRC mice model. (**A**) Tumor size in nude mice at the end of the study; (**B**) body weight growth curve of nude mice; (**C**) tumor weight in nude mice at the end of the study; (**D**) tumor growth curve in nude mice; (**E**) H&E staining of heart, liver, spleen, lung, and kidney in nude mice; (**F**,**G**) IHC staining and analysis of H&E, TUNEL, and Ki67 in mouse tumors (compared to the control group, * *p* < 0.05, *** *p* < 0.001). The red circle represents the area of necrosis in the tumor, and the red arrow indicates positive for Tunel and Ki67 staining.

To evaluate the antitumor effect of DHTS in vivo, HCT116/OXA tumor-bearing nude mice were constructed and treated with DHTS (40 mg/kg). The results showed that DHTS treatment markedly reduced the tumor weight and volume than that of the control group, and OXA had no significant inhibition on tumor growth (Figure 7A,C,D).

As shown in Figure 7B, no significant decrease in weight was observed after mice were treated with DHTS compared with the control group. H&E staining of the heart, liver, spleen, lung, and kidney in each group was performed, which demonstrated that DHTS had no toxicity in these organs (Figure 7E). In addition, H&E staining showed that the tumor necrosis area had a great increase in the DHTS group (Figure 7F,G). We further analyzed tumor proliferation by detecting TUNEL and Ki-67 through IHC staining. As demonstrated in Figure 7F,G, treatment with DHTS elevated the apoptosis of HCT116/OXA cells by the TUNEL assay and significantly declined Ki-67 expression compared to the control group.

**Figure 8:**
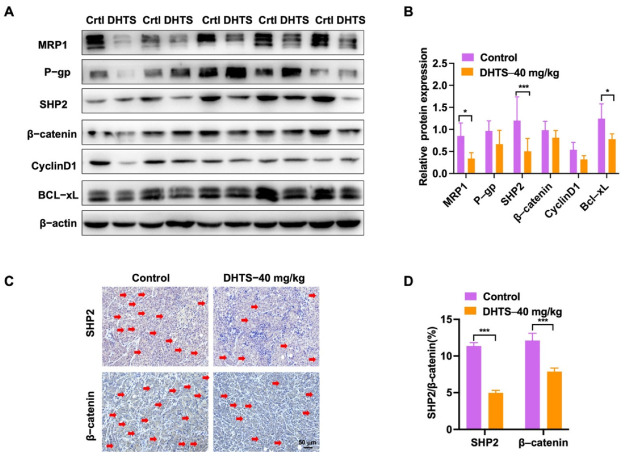
DHTS reduces the expression of drug resistance, apoptosis-related proteins, SHP2, and Wnt/β-catenin in tumor tissues between the control group and DHTS group. (**A**,**B**) Protein expression and data statistics of MRP1, P-gp, SHP2, β-catenin, Cyclin D1, and Bcl-xL by western blot; (**C**,**D**) representative images of IHC staining and data statistics of SHP2 and β-catenin (compared to the control group, * *p* < 0.05, *** *p* < 0.001). The red arrow indicates positive for SHP2 and β- Catenin staining.

In addition, we analyzed the change in drug-resistant related proteins by western blot. The results showed that the DHTS group could significantly inhibit protein expression of MRP1, SHP2, and Bcl-xL compared with the control group (Figure 8A,B). SHP2 and β-catenin expressions were also decreased in the DHTS group through IHC staining (Figure 8C,D). Thus, DHTS has antitumor activity in vitro and in vivo against OXA-resistant CRC.

## 3. Materials and Methods

### 3.1. Virtual Screening

With the structure of a known compound as a query, ligand-based virtual screening (abbr. LBVS) can be used to identify different compounds that are similar to the query structure at a 2D or 3D level. We used KNIME [22] to compute the 2D similarity between 2D structures. To compute the 3D similarity, multiple 3D conformations of each compound were generated with the ETKDG algorithm [23] in RDkit [24], and then the Open3DALIGN [25] algorithm in RDKit was used to compute the 3D similarity between two 3D structures based on the overlap of their pharmacophores and heavy atoms (Figure S2).

We first searched the Chinese Pharmacopoeia 2015 edition (National Commission of Chinese Pharmacopoeia, 2015) to find traditional Chinese medicines with high clinical application value and the related active components for the treatment of colorectal cancer. Then, we searched PubMed and GeenMedical using these active components: “Colorectal Cancer”, “HCT116”, “HT-29”, and “SW480”. These were used as search keywords to select compounds with anti-CRC effects in vitro or in vivo. Finally, 21 natural compounds (Table S3) were identified as the starting query molecules. To identify novel scaffold compounds, these 21 compounds were used to screen the MCE natural product database (containing 6863 compounds) by 3D similarity. Compounds with 3D similarity greater than 0.7 were kept for a 2D similarity check. To ensure the scaffold novelty and good druggability, the compounds with a 2D similarity less than 0.5 and molecular weight less than 700 were selected.

### 3.2. Cell Culture

The human colorectal carcinoma HCT116 cells were purchased from the Chinese Academy of Sciences (Shanghai, China). HCT116 cells were cultured in McCoy’s 5A Modified Medium (Gibco, Grand Island, NY, USA) supplemented with 10% FBS (YEASEN, Shanghai, China) and 1% penicillin-streptomycin (YEASEN, Shanghai, China) in a 5% CO_2_ incubator at 37 °C. HCT116/OXA cells were constructed by stepwise, continuous treatment with OXA as previously described [26]. Initially, 100 ng/mL OXA (Meilunbio, Dalian, China) was used to induce drug resistance in HCT116 cells, and then the concentration of OXA was gradually increased. After about 8 months, the cells could stably grow in 10 μg/mL OXA and be named HCT116/OXA cell line.

### 3.3. Cell Counting Kit-8 (CCK-8) Assay

CCK-8 assays were used to detect the inhibitory activity of DHTS on the proliferation of HCT116 or HCT116/OXA cells. Cells were added to the 96-well plate and cultured for about 12–16 h after cell adherence; having carefully discarded the medium with a pipette discharge to avoid laceration of cells. Then, the cells were treated with different concentrations of the test compounds. After treatment for 24 or 48 h, cell viability was determined using CCK-8 (Meilunbio, Dalian, China) following the manufacturer’s instruction.

### 3.4. Calcein Acetoxymethyl Ester/Propidium Iodide (Calcein-AM/PI) Staining

Cells in 96-well plates were incubated with various doses of DHTS for 24 or 48 h. A Calcein-AM/PI dual staining kit (Meilunbio, Dalian, China) with two fluorescent dyes was used to simultaneously detect live and dead cells. As Calcein-AM is converted to calcein, living cells produce green fluorescence, while dead cells produce red fluorescence due to the presence of PI. Live and dead cells were detected and analyzed by the Molecular Devices (Operetta CLS, PerkinElmer, Waltham, MA, USA) according to the manufacturer’s instructions.

### 3.5. Apoptosis Analysis

Cells were collected after 24 or 48 h of treatment with OXA or DHTS. An apoptosis kit (Meilunbio, Dalian, China) was used to detect apoptosis according to the manufacturer’s instructions. Cells were harvested and washed with PBS and incubated in Annexin V-FITC and PI solution for flow cytometry assay (BD Biosciences, Franklin Lakes, NJ, USA).

### 3.6. Cell Cycle Analysis

The cell cycle was detected using cell cycle kits (Meilunbio, Dalian, China). The cells were cultured and treated with OXA or DHTS of various concentrations for 24 or 48 h. The cells were collected and fixed in 70% ethanol at 4 °C overnight. After fixation, the cells were centrifuged at 1000 rpm for 3 min. Cells were stained with PI/RNase A solution (5 μg/mL PI and 100 mg/mL RNase A) at 37 °C in the dark for 30 min. The phase distribution of the cell cycle was detected using a flow cytometer.

### 3.7. Fluorescence Assay

After the cells were treated with OXA or DHTS for 48 h, γ-H2AX (#T55269, Abmart, Shanghai, China) and Alexa Fluor 488-conjugated Goat Anti-Rabbit IgG (H+L) (#AS053, Abclonal, Wuhan, China) for labeling and DAPI (#C1005, Beyotime, Shanghai, China) for staining the nucleus were used according to the instructions of the manufacturer. DNA damage in cells was imaged and analyzed using a high-resolution microscopy (GE, OMX SR, Fairfield, CT, USA).

### 3.8. Western Blotting

The cells were collected and lysed in ice-cold RIPA buffer containing protease inhibitors. Then, the total protein was quantified using a BCA assay kit (#P0010s, Beyotime, Shanghai, China). The protein was then separated in SDS-PAGE and transferred to the PVDF membrane (#10600021, GE, Fairfield, CT, USA). Then, 1% Tween-20 (TBST, pH 7.4) with 5% non-fat powdered milk for blocking was used and the primary antibody was incubated at 4 °C for 16 h. A primary antibody consists of: MRP1, Bcl-xL, Bcl-2, SHP2, β-catenin, Cyclin D1(#14685, #2764s, #3498S, #3397, #8480, #55506, Cell Signaling Technology, Danvers, MA, USA), P-gp (#ab170904, Abcam, Boston, MA, USA), GAPDH, and β-actin (#60004-1, #66009-1, Proteintech, Wuhan, China). After washing three times with TBST, the membrane was incubated with the secondary antibody. A secondary antibody consists of HRP-conjugated Affinipure Goat Anti-Mouse IgG (H+L) and HRP-conjugated Affinipure Goat Anti-Rabbit IgG (H+L) (#SA00001-1, #SA00001-2, Proteintech, Wuhan, China). The gels were visualized under a gel imaging system (Tanon, Shanghai, China) and Image J software (version 1.52, NIH, Washington, DC, USA) was used to measure and analyze the density of the band.

### 3.9. Animal Experiment

Male BALB/C nude mice (4 weeks old) were purchased from Shanghai Jihui Experimental Animal Co., Ltd. HCT116/OXA cells (5 × 10^6^ cells per mouse) were subcutaneously injected into the left side of nude mice. Tumors grown in mice were removed, evenly cut into tumors of about 15 mm^3^ in size, and inserted into the armpits of nude mice. When the tumor volume reached about 50 mm^3^, the mice were randomly divided into three groups: a control group, OXA (5 mg/kg, i.p.), and DHTS (40 mg/kg, i.p.). The OXA group was formulated with PBS as a solvent, and the control and DHTS groups were formulated with 5% DMSO, 30% PEG-400, and 65% PBS as solvents. OXA was administered every three days. Control and DHTS groups were daily administered for 14 days. The tumor length (L) and width (W) were obtained by caliper, and tumor volumes were calculated using the formula: L × (W)^2^/2. Two weeks later, the mice were sacrificed, and tumor and viscera were subjected to hematoxylin and Eosin (H&E), as well as immunohistochemistry (IHC) staining of TUNEL, Ki-67, SHP2, and β-catenin expression. All procedures involving animals were performed in accordance with the ethical standards for the care and use of laboratory animals and related ethical regulations (PZSHUTCM210913005) of the Shanghai University of Traditional Chinese Medicine.

### 3.10. Statistical Analysis

Statistical analysis was conducted using GraphPad Prism 8.0 software. Data were presented as means ± standard deviation (SD). All experiment data were statistically evaluated by using a two-tailed student’s *t*-test or one-way ANOVA with Tukey’s multiple comparison tests. Values of *p* < 0.05 were considered significant.

## 4. Discussion

Colorectal screening programs and OXA-based first-line chemotherapy are associated with improved overall survival of CRC [27]. However, the acquired resistance of CRC to OXA is the main cause of clinical treatment failure. Hence, it is necessary to identify a novel therapeutic strategy to overcome OXA resistance. Different from random screening at the cellular level, we intended to try a new method of quickly finding active compounds from natural products, so we performed ligand-based virtual screening through selecting known compounds with anti-CRC effects as query molecules. First, we searched the Chinese Pharmacopoeia 2015 edition (National Commission of Chinese Pharmacopoeia, 2015) to find the traditional Chinese medicines and the related active components for the treatment of colorectal cancer; this information has higher clinical application value than directly searching the active compounds from the literature. Second, we wanted to identify novel scaffold compounds, and so we set the following criteria: (1) compounds with 3D similarity greater than 0.7; and (2), to ensure the scaffold novelty and good druggability, the compounds with 2D similarity less than 0.5 and molecular weight less than 700 were selected. Importantly, it depended on the structure and quantity of the screening library as to whether we could screen the novel scaffold compounds. Thus, ligand-based virtual screening containing 21 natural compounds with anti-CRC activities and structural diversity was applied to discover the new active compounds against CRC. Interestingly, 48 compounds were screened out and 15 compounds were used for determining their inhibitory effects on the proliferation of HCT116 cells. The results showed that DHTS had the strongest inhibitory activity against HCT116 cell proliferation among these compounds. Therefore, it is a sufficient method by which to discover the active compounds through ligand-based virtual screening; 2 compounds (DHTS and Luteolin) out of the 15 showed inhibition rates lower than 50% at 5 μM. Given the active ingredients from *Salvia miltiorrhiza* have anti-tumor activities [28,29,30], we bought another 8 compounds from *Salvia miltiorrhiza* for determining their anti-CRC activities. The results showed that the inhibition rate of DHTS on the proliferation of HCT116 cells was significantly higher than that of the other 8 active ingredients of *Salvia miltiorrhiza*. Therefore, DHTS was selected for further study against CRC.

DHTS is a tanshinone analog with antitumor effects, including amelioration of tumor multidrug resistance problems [31,32]. For breast cancer, DHTS reduced the expression of the anti-apoptotic protein Bcl-xL in MCF-7 and MDA-MB-231 cells [33]. In addition, DHTS inhibits the growth of osteosarcoma by the Wnt/β-catenin signaling pathway [13]. DHTS also decreased the expression of LRP6 (upstream of β-catenin), β-catenin, c-Myc (downstream of β-catenin), and Cyclin D1 proteins levels to inhibit Wnt/β-catenin signaling [13]. In CRC, DHTS can enhance the cytotoxicity of doxorubicin and irinotecan in P-gp overexpressing SW620 Ad300 colon cancer cells by reducing P-gp mRNA and protein levels, and by inhibiting P-gp ATPase activity [32]. Studies also showed that DHTS exhibits anticancer activity in a HCT116 xenograft nude mouse model [12], whereas there are no studies on the effect of DHTS on HCT116/OXA cells and animals.

**Figure 9:**
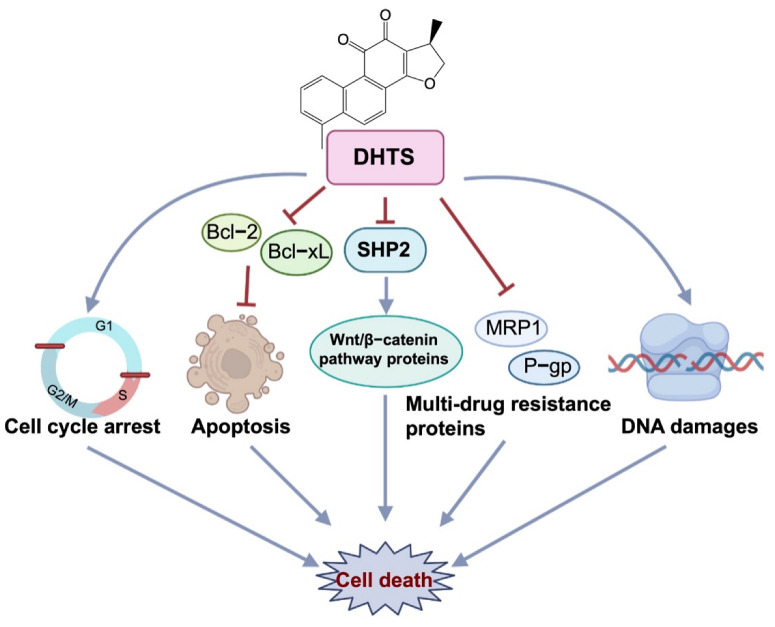
Antitumor effect of DHTS on OXA-resistant CRC. DHTS can exert anti-OXA-induced CRC resistance through multiple links and targets, including blocking the cell cycle in S and G_2_/M phases, promoting apoptosis, inducing DNA damage, and reducing MRP1, P-gp, Bcl-2, Bcl-xL, SHP2, and β-catenin protein.

To investigate whether DHTS has anti-OXA-resistant CRC effects in vitro and in vivo, we constructed OXA-resistant HCT116/OXA cells and the corresponding tumor-bearing nude mice model for the study. DHTS could inhibit the growth of OXA-resistant CRC in vitro and in vivo. In HCT116/OXA cells, DHTS significantly inhibited cell proliferation, induced cell apoptosis, blocked cell cycle in S and G_2_/M phases, and enhanced DNA damage in a concentration-dependent manner. MRP1 and P-gp proteins are the main drug efflux transporters responsible for treatment failure in many cancers [34,35]. Previous studies have reported that both mRNA and protein levels of MDR markers, including P-gp, MRP2, and breast cancer resistance protein (BCRP), were significantly higher in HCT116/OXA cells than in HCT116 cells [36]. In agreement with the study, we found that the expression levels of MRP1 and P-gp proteins in HCT116/OXA cells were also significantly increased. In addition, SHP2 and Wnt/β-catenin-associated signaling pathways are related to OXA-induced CRC drug resistance [37,38]. Therefore, we investigated the expression levels of drug-resistant proteins MRP1 and P-gp, anti-apoptotic proteins Bcl-xL, and SHP2, β-catenin, and Cyclin D1. The results showed that the expression of drug resistance proteins MRP1 and P-gp, anti-apoptotic proteins Bcl-2, and Bcl-xL decreased after treatment with DHTS in HCT116/OXA cells and tumor tissues. Therefore, DHTS inhibits the growth of the drug-resistant HCT116/OXA strain and HCT116/OXA xenograft tumor through multiple aspects, including promoting apoptosis, blocking cell cycle in S and G_2_/M phases, inducing DNA damage, and reducing the expression of drug-resistant proteins MRP1 and P-gp (Figure 9).

Taken together, this study is the first to reveal that DHTS inhibits tumor growth in OXA-resistant HCT116 CRC in vitro and in vivo. In view of the essential role of SHP2 in carcinogenic KRAS-mutation-driven CRC tumors, the mechanism of DHTS targeting SHP2 to overcome CRC resistance to OXA should be further investigated. In addition, the aqueous solubility and biocompatibility of DHTS are rather low, which impedes its biological applications to a large extent [39]. Owing to its low molecular weight, DHTS is suitable for further structural optimization to improve its solubility and biocompatibility. Moreover, DHTS had excellent biosafety in animal models. Similar to previous results [40], our present study also demonstrated that no nude mice died during the treatment periods in the DHTS group, and no obvious normal tissue toxicity in the heart, liver, spleen, lung, or kidney was observed in nude mice (Figure 7E). Therefore, DHTS has the potential to be developed for sensitizing resistant cancer cells or to be used as a novel lead compound to improve the therapeutic efficacy for OXA-resistant CRC.

## Data Availability

The data that support the findings of this study are available from the corresponding author upon reasonable request.

## Supplementary Materials

The following supporting information can be downloaded at: https://www.mdpi.com/article/10.3390/molecules27227774/s1, Figure S1: LC-MS of Dihydrotanshinone I; Figure S2: Flow chart of virtual screening; Table S1: Fifteen compounds obtained from LBVS; Table S2: Eight compounds from *Salvia miltiorrhiza*; Table S3: Twenty-one known natural compounds with anti-CRC effects.

## Sample Availability

Not available.
