# Supplementary material for: Dihydrotanshinone I Inhibits the Proliferation and Growth of Oxaliplatin-Resistant Human HCT116 Colorectal Cancer Cells
Source: Molecules. 2022 Nov 11;27(22):7774. doi: 10.3390/molecules27227774 (PMC9692243; doi:10.3390/molecules27227774)
Supplement: Supplementary file 1 [file molecules-27-07774-s001.zip › molecules-1987027-SI.pdf]

Supplementary material

Dihydrotanshinone I inhibits the proliferation and growth of Oxaliplatin-resistant human HCT116 colorectal cancer cells

Mengge Wang[1], Yusen Xiang[1], Ruyu Wang[1], Lijun Zhang[1], Hong Zhang[1], Hongzhuan Chen[1], Xin Luan[1,*], Lili Chen[1,*]

[1]Shanghai Frontiers Science Center of TCM Chemical Biology, Institute of Interdisciplinary Integrative Medicine Research, Shanghai University of Traditional Chinese Medicine, Shanghai, 201203, China

*Correspondence:

Xin Luan, Ph D

Shanghai University of Traditional Chinese Medicine, Shanghai 201203, China

Tel./fax: +86 21 51322428

E-mail: luanxin@shutcm.edu.cn

Lili Chen, Ph D

Shanghai Frontiers Science Center for Chinese Medicine Chemical Biology, Institute of Interdisciplinary Integrative Medicine Research, Shanghai University of Traditional Chinese Medicine, Shanghai, 201203, China

Tel./fax: +86 21 51322720

E-mail: llchen@shutcm.edu.cn

Table S1 Fifteen compounds obtained from LBVS.

Number	Name	Compound structure
1	Dihydrotanshinone I	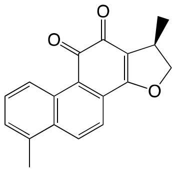
2	Chrysin	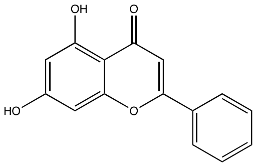
3	Doxycycline hyclate	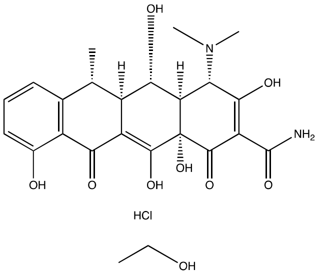
4	Sodium erythorbate	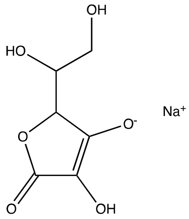
5	Luteolin	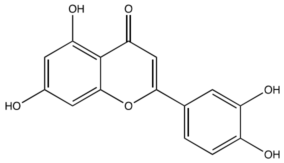
6	Xanthine	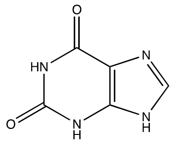
7	Taxifolin	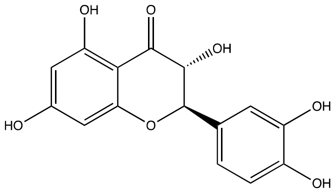

8	Prednisone acetate	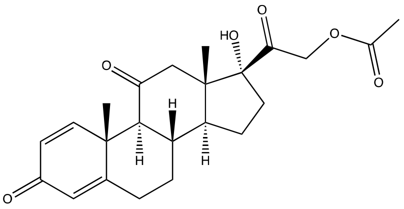
9	Ftaxilide	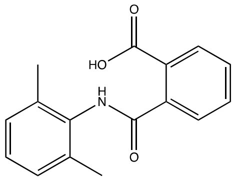
10	7,4'-dihydroxyflavone	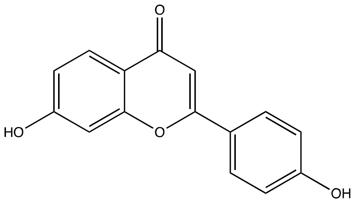
11	Allantoin	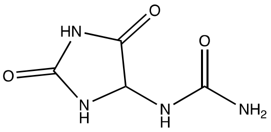
12	L-Dihydroorotic acid	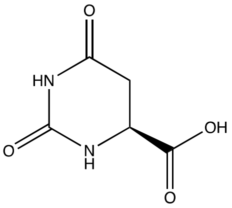
13	Dehydrocholic acid	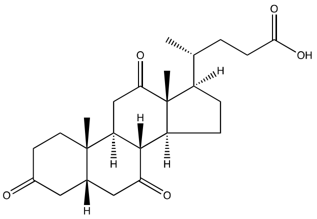
14	Deoxycorticosterone acetate	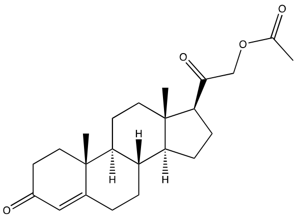
15	Theobromine	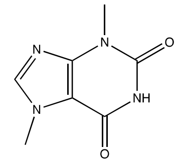

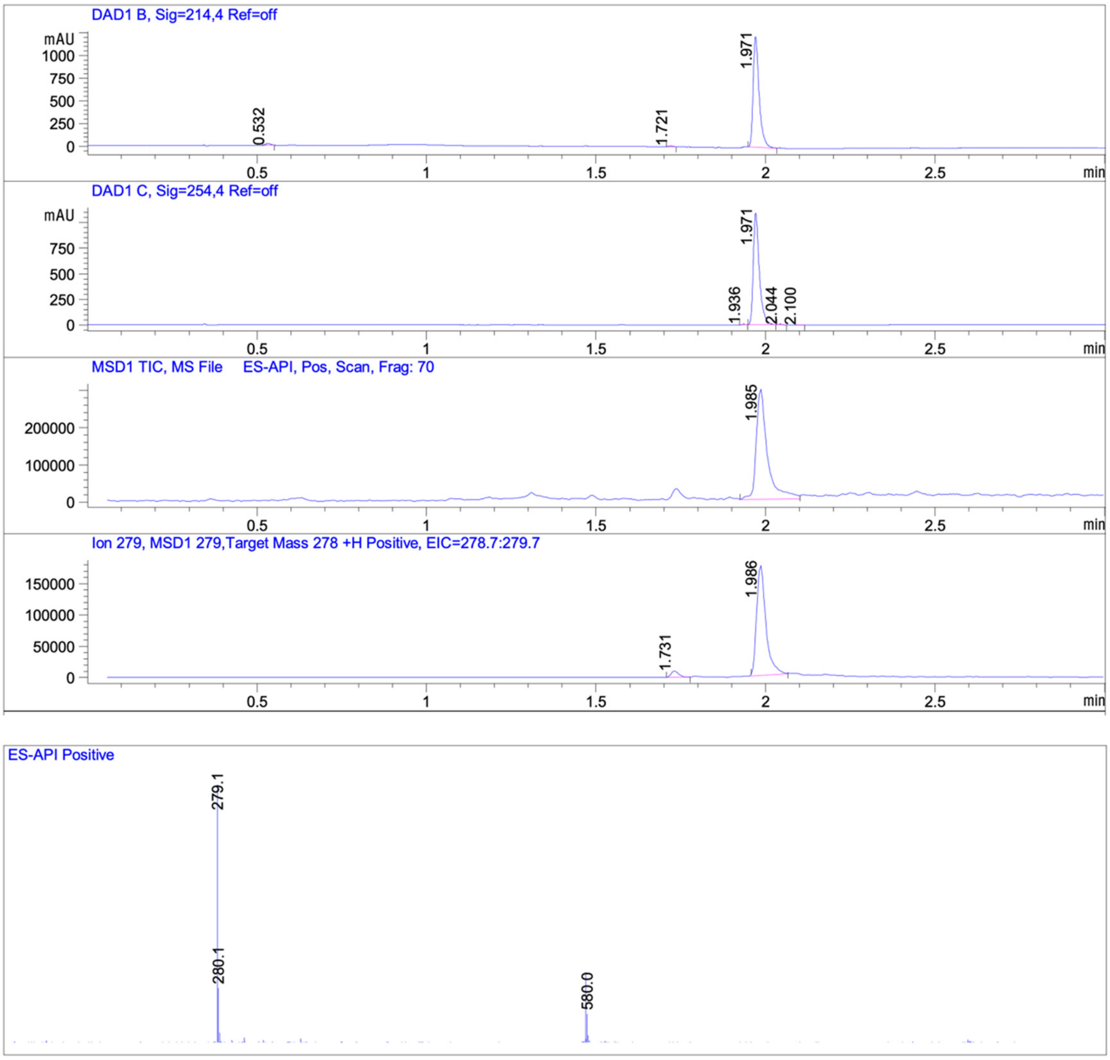

Figure S1 LC-MS of Dihydrotanshinone I

Table S2 Eight compounds from *Salvia miltiorrhiza*.

Number	Name	Compound structure
1	Cryptotanshinone	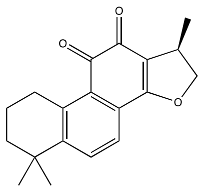
2	Danshensu	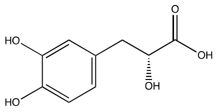

3	Danshensu sodium salt	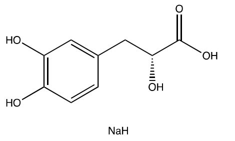
4	Tanshinone I	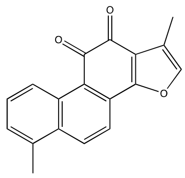
5	Tanshinone IIA	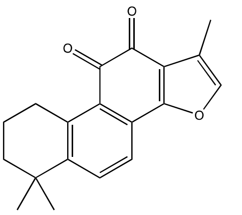
6	Tanshinone IIA sulfonate sodium	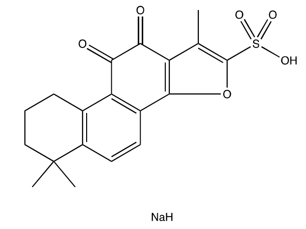
7	Lithospermic acid	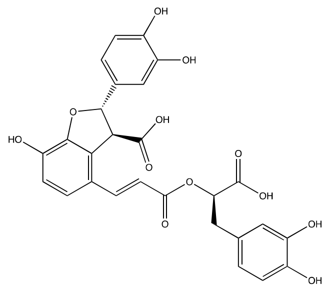
8	Salvianolic acid B	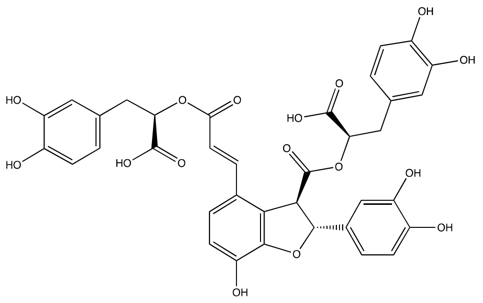

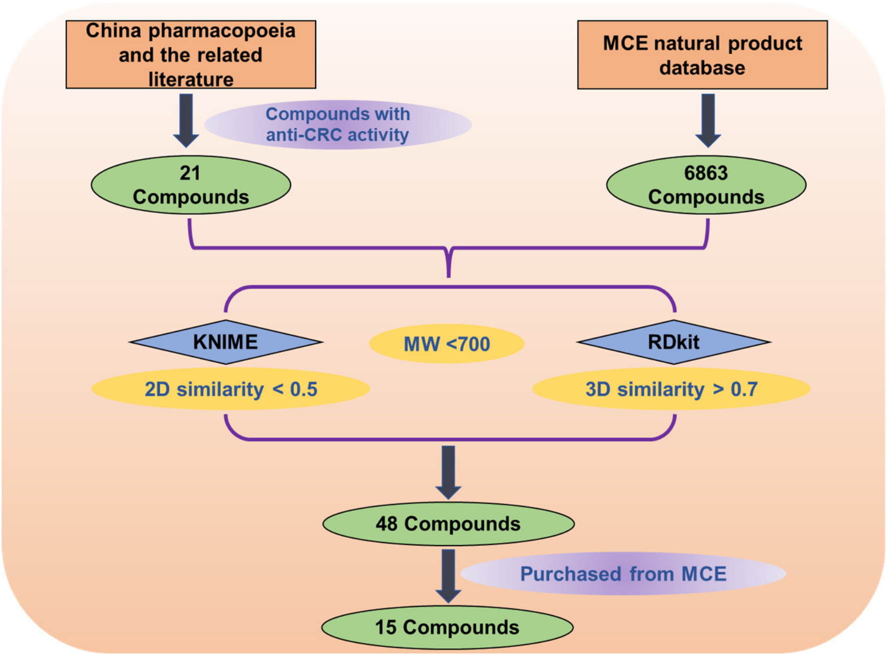

Figure S2 Flow chart of virtual screening. First, 21 natural compounds with anti-CRC activity were selected from the Chinese Pharmacopoeia and related literature. Then, 21 compounds were used as query structures to screen the MCE natural product database (6863 compounds) by 2D and 3D similarity. KNIME was used to compute the 2D similarity between 2D structures. 3D conformations of each compound were generated with ETKDG algorithm in RDkit, then Open3DALIGN algorithm in RDKit was used to compute the 3D similarity between two 3D structures based on the overlap of their pharmacophores and heavy atoms. 48 compounds with 2D similarity less than 0.5 and 3D similarity greater than 0.7 and molecular weight less than 700 were selected. Finally, 15 compounds were purchased from MCE to determine their inhibitory effects on the proliferation of HCT116 cells.

Table S3 Twenty-one known natural compounds with anti-CRC effects.

Number	Name	Compound structure

1	Frangula emodin	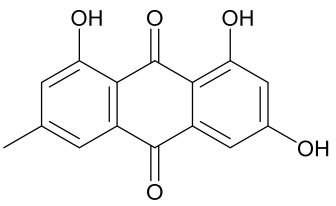
2	Rhabarberone	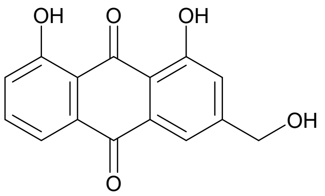
3	Esculentoside A	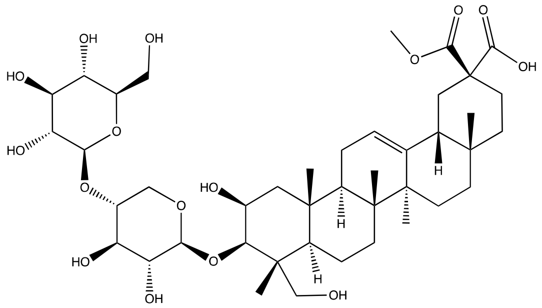
4	Tripterin (Celastrol)	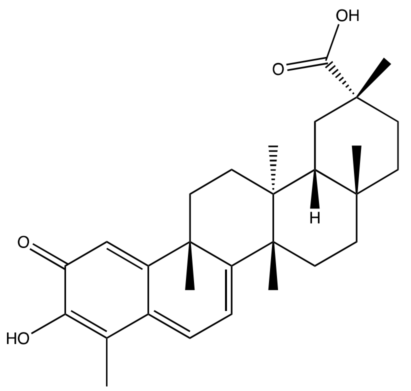
5	Honokiol	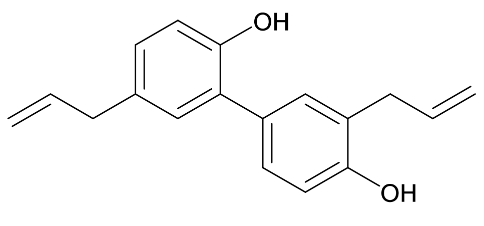
6	Resveratrol	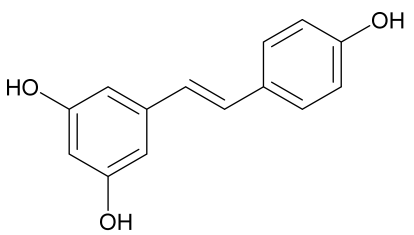

7	Evodiamine	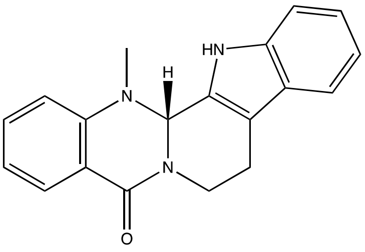
8	Octahydrocurcumin	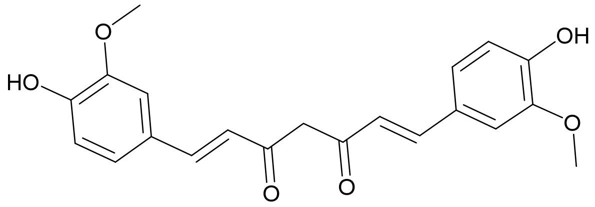
9	Tanshinone IIA	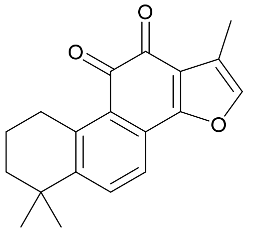
10	Cryptotanshinone	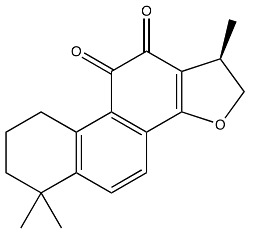
11	Arctiin	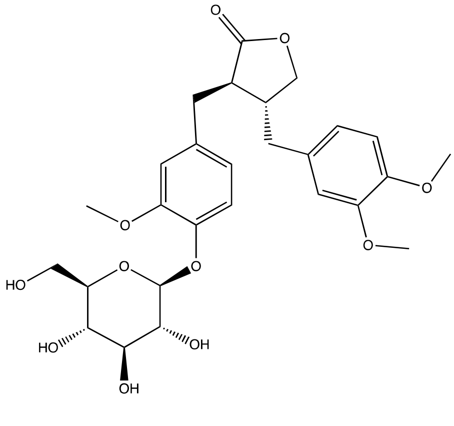
12	Berberine	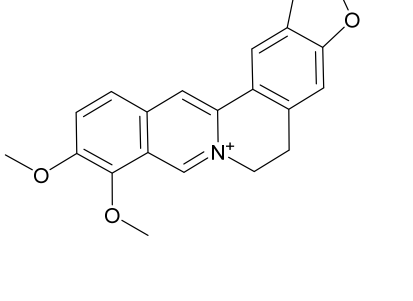

13	Andrographolide	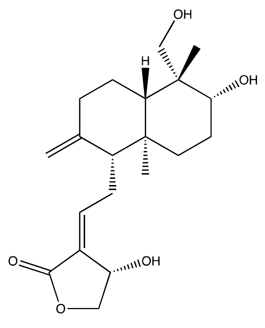
14	Polyphyllin I	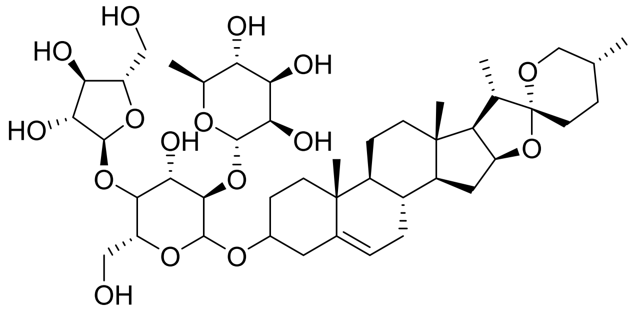
15	cantharidin	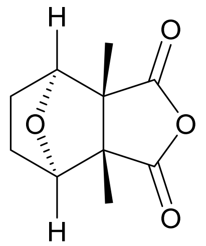
16	Norcantharidin	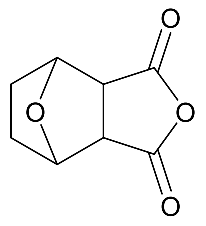
17	Sodium Cantharidinate For Injection	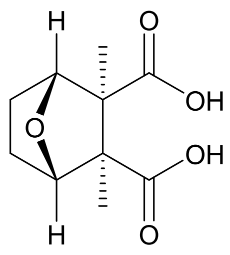

18 GypenosideA

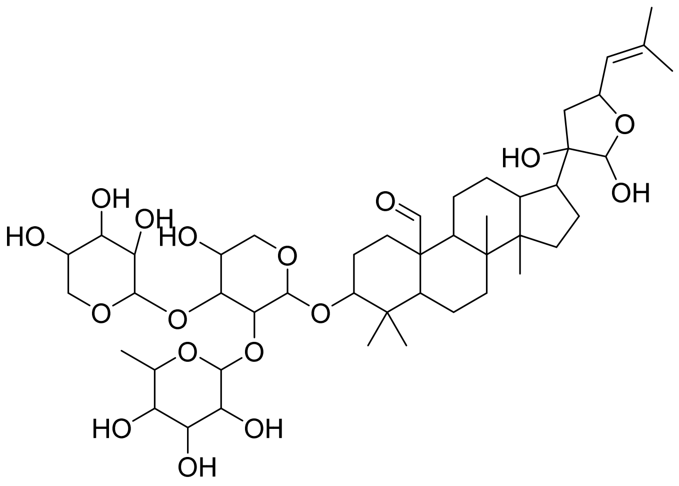

19 GypenosideXLIX

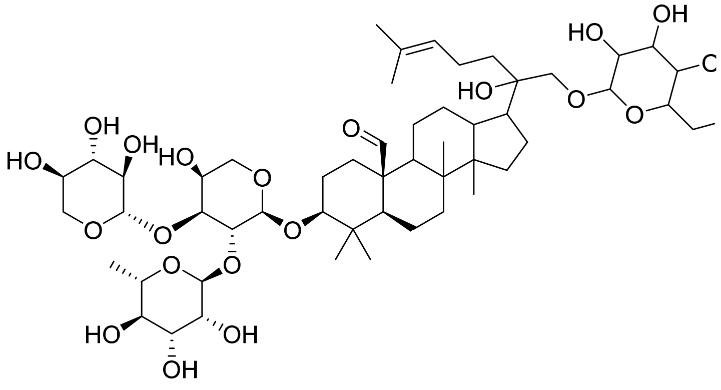

20 GypenosideXLVI

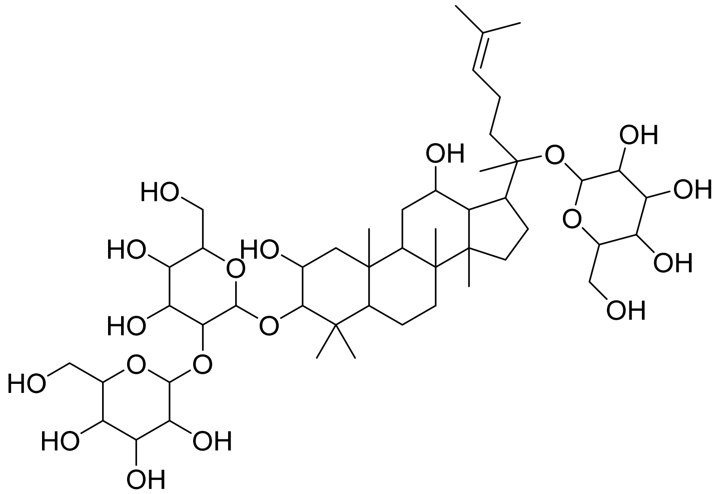

21	GypenosideXIII	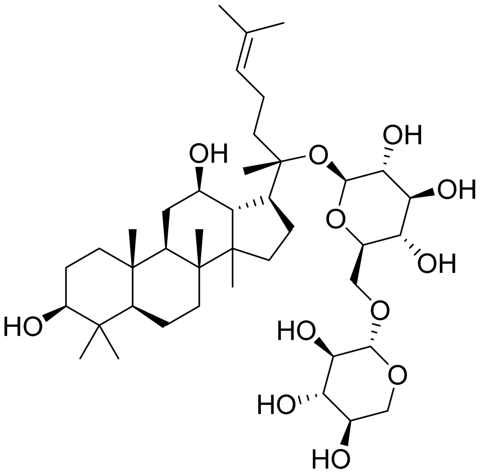
